# The Fermented Soy Product ImmuBalance^TM^ Suppresses Airway Inflammation in a Murine Model of Asthma

**DOI:** 10.3390/nu13103380

**Published:** 2021-09-26

**Authors:** Hideaki Kadotani, Kazuhisa Asai, Atsushi Miyamoto, Kohei Iwasaki, Takahiro Kawai, Misako Nishimura, Mitsunori Tohda, Atsuko Okamoto, Kanako Sato, Kazuhiro Yamada, Naoki Ijiri, Tetsuya Watanabe, Tomoya Kawaguchi

**Affiliations:** Department of Respiratory Medicine, Graduate School of Medicine, Osaka City University, Osaka 545-8585, Japan; h-kadotani@med.osaka-cu.ac.jp (H.K.); atsush1@med.osaka-cu.ac.jp (A.M.); k.iwasaki@med.osaka-cu.ac.jp (K.I.); t-kawai@med.osaka-cu.ac.jp (T.K.); nishimura.misako@med.osaka-cu.ac.jp (M.N.); m2073301@med.osaka-cu.ac.jp (M.T.); m1169499@med.osaka-cu.ac.jp (A.O.); m1162967@med.osaka-cu.ac.jp (K.S.); kazuhironishiyamato@gmail.com (K.Y.); m0021923@med.osaka-cu.ac.jp (N.I.); m2048518@med.osaka-cu.ac.jp (T.W.); kawaguchi.tomoya@med.osaka-cu.ac.jp (T.K.)

**Keywords:** fermented soy product, asthma, eosinophilic inflammation, Th2 cytokines

## Abstract

The fermented soy product ImmuBalance contains many active ingredients and its beneficial effects on some allergic diseases have been reported. We hypothesized that ImmuBalance could have potential effects on airway inflammation in a murine model of asthma. Mice sensitized and challenged with ovalbumin developed airway inflammation. Bronchoalveolar lavage fluid was assessed for inflammatory cell counts and levels of cytokines. Lung tissues were examined for cell infiltration and mucus hypersecretion. Oral administration of ImmuBalance significantly inhibited ovalbumin-induced eosinophilic inflammation and decreased Th2 cytokine levels in bronchoalveolar lavage fluid (*p* < 0.05). In addition, lung histological analysis showed that ImmuBalance inhibited inflammatory cell infiltration and airway mucus production. Our findings suggest that supplementation with ImmuBalance may provide a novel strategy for the prevention or treatment of allergic airway inflammation.

## 1. Introduction

The prevalence of allergic diseases such as asthma, rhinitis, atopic dermatitis, and food allergy, has increased globally in recent years [1,2,3,4]. Asthma is one of the most common allergic diseases, and is usually characterized by chronic airway inflammation. Asthma is defined by respiratory symptoms such as wheezing, shortness of breath, chest tightness, and cough that vary over time and intensity, with variable expiratory airway limitations (Global Initiative for Asthma (GINA) 2021). In patients with allergic asthma, an imbalance in lymphocyte immunity, especially between Th1 and Th2 responses, leads to immunoglobulin E (IgE) production, T cell and eosinophil infiltration, airway hyperresponsiveness, mucus cell hyperplasia, and airway remodeling, resulting in the appearance of asthma symptoms [5]. Therefore, regulation of the balance between Th1 and Th2 responses is key to controlling allergic asthma.

In clinical practice, inhaled corticosteroids are the basis of asthma treatment regardless of the phenotype. Moreover, the administration of systemic corticosteroids is still important for patients with exacerbations or who have severe asthma. However, adverse events from corticosteroid treatment have been reported [6,7,8]. In addition to corticosteroids, bronchodilators, and anticholinergics are also used for treating asthma, though they do not improve the nature of asthma. Therefore, alternative treatments or prevention methods are required. Previous studies reported that dietary soy consumption is associated with lung function and symptoms in asthma patients or prevalence of allergic rhinitis [9,10]. Based on these studies, we decided to study the effects of a soy product on allergic airway inflammation.

Recently, it was reported that imbalances in the gut microbiota composition, described as dysbiosis, may be involved in immune system and allergic diseases [11]. Commensal gut microbiota synthesizes short-chain fatty acids (SCFAs), such as acetate, butyrate, and propionate, by fermenting dietary fiber. Some studies have shown that SCFAs have beneficial effects in an allergic asthma model [12,13,14,15] and that butyrate inhibited the migration of eosinophils to the lungs or airways, inducing apoptosis [16]. In addition, other studies have reported that the intake of soy protein changes the gut microbiota and increases the concentration of SCFAs in the feces [17,18].

ImmuBalance^TM^ (Nichimo Biotics Co., Ltd., Tokyo, Japan), the name of which comes from its ability to modulate the balance of the immune system, is a fermentation product made from defatted soybeans with *Aspergillus oryzae* and lactic acid bacteria (*Pediococcus parvulus* and *Enterococcus faecium*) according to a proprietary fermentation technology. Koji mold, *A. oryzae*, is designated as a national microorganism of Japan as it is essential for producing several traditional Japanese food products, such as soy sauce, miso, and sake [19]. A pilot study reported that the administration of ImmuBalance improved the clinical symptoms of cedar pollinosis in Japan [20]. In animal models, some experiments have been conducted on the effectiveness of ImmuBalance as an anti-allergic food, and it was reported that ImmuBalance improved the symptoms of atopic dermatitis [21] and peanut allergy [22]. Moreover, another study reported that ImmuBalance alleviated chronic kidney disease through an anti-inflammatory effect [23]. However, the effect of ImmuBalance on allergic airway inflammation, particularly in asthma, has not been reported. Based on these previous studies, we hypothesized that ImmuBalance may have a beneficial effect in a murine model of allergic asthma and that SCFAs may be involved in the effect.

## 2. Materials and Methods

### 2.1. Animals

Seven-week-old BALB/c female mice (14.5 to 17.5 g) were obtained from Japan SLC (Shizuoka, Japan), and maintained under pathogen-free conditions at a controlled temperature of 23 ± 2 °C and a 12 h light/dark cycle with free access to water and diet. The animals were acclimated to the environment for one week before experiments began. The animal experiments were approved by the Ethics Committee of the Institutional Animal Care and Use of Osaka City University Graduate School of Medicine (Permit Number: 20009, 25 June 2020). All animal experiments were conducted in accordance with the Regulations on Animal Experiments in Osaka City University following the Guidelines for Proper Conduct of Animal Experiments in Japan.

### 2.2. Sensitization and Airway Challenge Protocol

**Figure 1:**
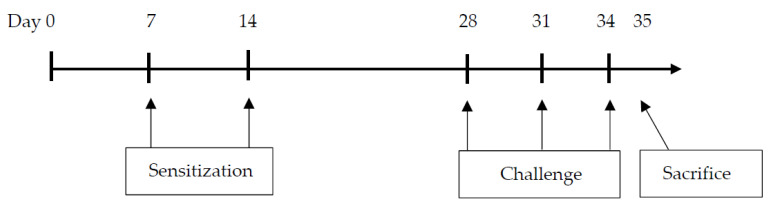
Experimental protocol. All mice were sacrificed on day 35 (24 h after the last challenge).

The mice were divided into four groups (n = 8) as follows: (1) phosphate-buffered saline (PBS)-sensitized/challenged + standard diet group (Control group); (2) PBS-sensitized/challenged + ImmuBalance-containing diet group (IMB group); (3) ovalbumin (OVA)-sensitized/challenged + standard diet group (OVA group); and (4) OVA-sensitized/challenged + ImmuBalance-containing diet group (OVA/IMB group). Mice in the OVA and OVA/IMB groups were sensitized by the intraperitoneal injection of 50 µg of OVA (Grade V; Sigma-Aldrich, St. Louis, MO, USA) with 1 mg of aluminum hydroxide (Imject Alum; Pierce Biotechnology Inc., Rockford, IL, USA) in 200 µL of PBS on days 7 and 14. Mice were challenged via the airways with OVA (1% *w/v* diluted in PBS) for 20 min using an ultrasonic nebulizer (NE-U780; Omron, Tokyo, Japan) on days 28, 31, and 34. The mice in the other groups (Control and IMB groups) were sensitized and challenged with PBS alone in the same manner. On day 35, 24 h after the last inhalation of OVA, the mice were sacrificed under inhalation anesthesia with isoflurane. The experimental protocols are described in Figure 1.

### 2.3. Diets

CLEA Rodent Diet CE-2 was used as the standard diet (CLEA Japan, Inc., Tokyo, Japan). The mice in the IMB and OVA/IMB groups were fed a diet containing 1.0% (*w/w*) ImmuBalance added to the standard diet. Both diets were sterilized by gamma irradiation from a Co^60^ source at 15 kGy, and were prepared by CLEA Japan, Inc. ImmuBalance administration was started at the beginning of the experiment, before the asthma model was induced.

### 2.4. Bronchoalveolar Lavage Fluid (BALF) Analysis and Measurement of Cytokine Levels

The mice were tracheotomized and cannulated, then the lung was lavaged three times with 0.5 mL of PBS for the collection of BALF. The recovered BALF was centrifuged at 2500 rpm and 4 °C for 10 min, and the supernatant was collected. The cell pellet was resuspended in 1 mL of PBS, and subjected to a cytospin procedure using a Shandon Cytospin 3 centrifuge (Shandon Scientific Co., London, UK). The slides were stained with Giemsa, and the total cell counts and differential cell counts (macrophages, eosinophils, neutrophils, and lymphocytes) were determined according to the cellular morphology and staining characteristics. At least 200 cells were counted under 400× magnification in a blinded manner. The levels of the cytokines interleukin (IL)-4, IL-5, and interferon (IFN)-γ in BALF were measured by an ELISA Kit (R&D Systems, Minneapolis, MN, USA) according to the manufacturer’s instructions.

### 2.5. Analysis of Lung Tissue

The left lung was fixed with 10% formalin for 48 h at positive pressure (25 cmH_2_O), and embedded in paraffin. Next, 3 µm thick slices were stained by hematoxylin and eosin (HE) and periodic acid-Schiff (PAS) staining. A semi-quantitative scoring system was used to evaluate peribronchiolar and perivascular inflammation by HE staining, and goblet cell hyperplasia by PAS staining. Peribronchiolar and perivascular inflammation was graded by the severity of inflammatory cell infiltration using a 0- to 4-grade scoring system: 0, no cells; 1, a few cells; 2, a ring of cells 1 cell-layer deep; 3, a ring of cells 2 to 4 cell-layers deep; and 4, a ring of cells >4 cell-layers deep, as described previously [24]. Hyperplasia of goblet cells was assessed using a 0- to 4-grade scoring system: 0, no PAS-positive cells; 1, <25% PAS-positive cells; 2, 25% to 50% PAS-positive cells; 3, 50% to 75% PAS-positive cells; and 4, >75% PAS-positive cells, as described previously [25]. Scoring of inflammatory cells and goblet cells was performed in 10 different fields for each lung section under 200× magnification, and the average scores were calculated in a blinded manner.

### 2.6. Measurement of Serum OVA-Specific IgE

Collected blood from the cava vein was centrifuged at 10,000 rpm and 4 °C for 10 min, and the serum was stored at −80 °C. Values of serum OVA-specific IgE were determined with an OVA-Specific IgE ELISA Kit (Shibayagi, Gunma, Japan) according to the manufacturer’s instructions.

### 2.7. Measurement of SCFAs in the Cecum

SCFAs were quantified by liquid chromatography-mass spectrometry (LCMS-8060; Shimadzu Corporation, Kyoto, Japan). Feces were homogenized and freeze-dried to control the water content. Subsequently, aliquots (8 to 10 mg) were suspended in 0.3 mL of ethanol with an internal standard (2-ethylbutyric acid). The mixture was vortexed and sonicated for 5 min, then centrifuged at 15,000 rpm and 4 °C for 10 min. The supernatant was collected and derivatized with 1-ethyl-3-(3-dimethylaminopropyl) carbodiimide hydrochloride, 3-nitrophenylhydrazine, and pyridine in 75% methanol at room temperature for 30 min, with shaking. Aliquots were diluted with 75% methanol containing 0.5% formic acid, then subjected to liquid chromatography-mass spectrometry. Data acquisition and peak selection were performed using LabSolutions software (Shimadzu Corporation, Kyoto, Japan). The peak height value of each compound was normalized to that of the internal standard.

### 2.8. Statistical Analysis

Data are expressed as the mean ± standard error of the mean. Differences were evaluated with two-way analysis of variance with two factors (exposure: PBS or OVA; diet: standard diet or IMB) for multiple-group comparisons. A post hoc comparison was assessed with the Tukey–Kramer test when a significant interaction between exposure and diet was detected. Differences were considered to be significant when *p* < 0.05. All statistical analyses were performed with GraphPad Prism 7.04 (GraphPad Software, San Diego, CA, USA).

## 3. Results

### 3.1. Diet Consumption and Changes in Body Weight

**Figure 2:**
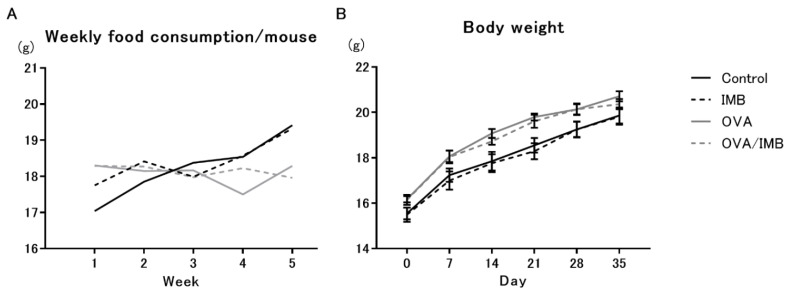
The amount of weekly food consumption (**A**) and the body weight changes (**B**) in each group. Values represent the mean ± standard error of the mean.

The daily consumption of diet was estimated by weekly measurements of the food remaining in the cages. There was no significant difference in food intake between the groups (2.5 to 3.0 g/mouse/day; Figure 2A). Body weights were measured on day 0 and every 7 days thereafter. On day 28 (initial challenge day), the body weights were measured before inhalation in consideration of the effect of the adherence of atomized particles on the body weight. The body weight and the rate of weekly weight gain did not differ between the groups (Figure 2B).

### 3.2. ImmuBalance Decreased the Number of Eosinophils and Other Inflammatory Cells in BALF

**Figure 3:**
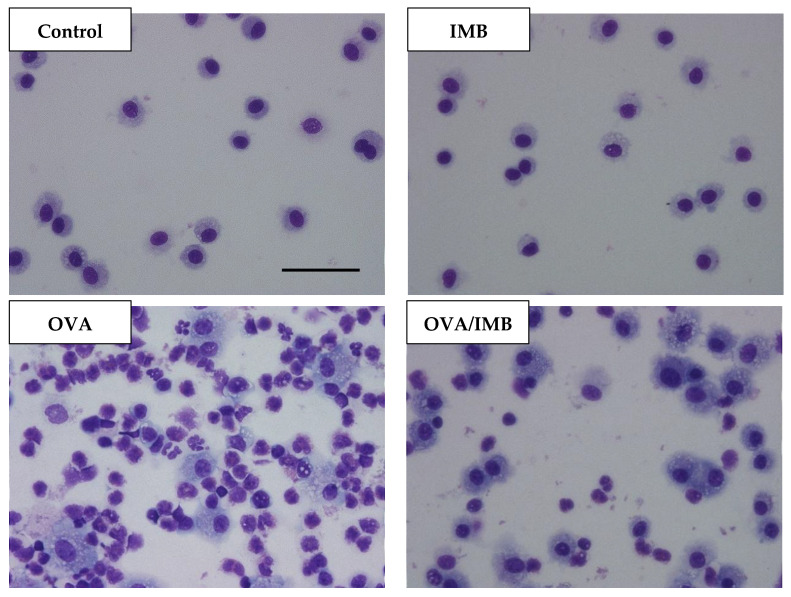
Representative images of BALF from each group are shown at 400× magnification. Scale bar = 50 µm.

**Figure 4:**
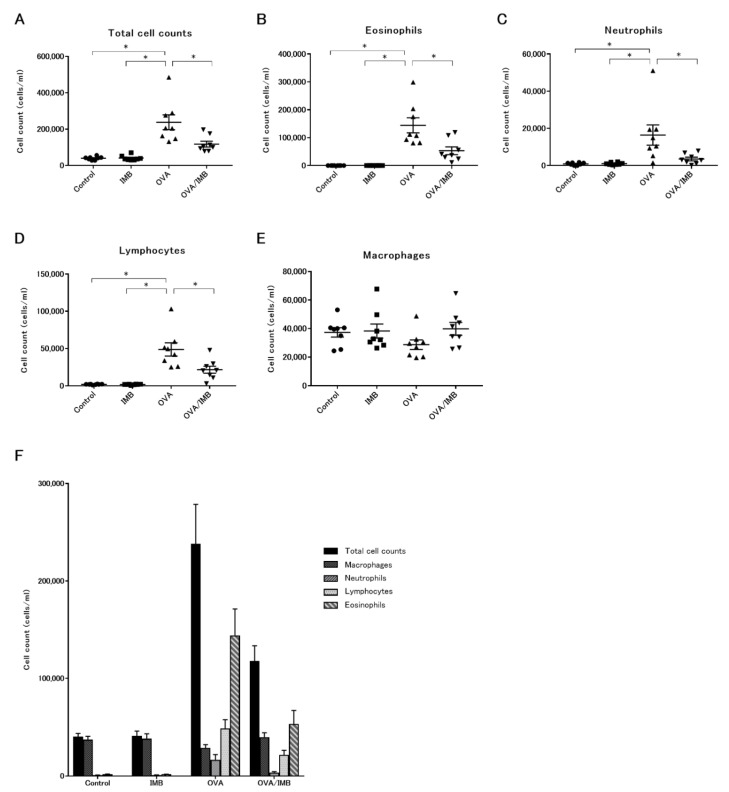
The total cell count and the number of eosinophils, neutrophils, and lymphocytes in BALF were significantly lower in the OVA/IMB group than in the OVA group, whereas the number of macrophages did not decrease. The numbers of total cells (**A**), eosinophils (**B**), neutrophils (**C**), lymphocytes (**D**), and macrophages (**E**). The cell fractions in each group (**F**). Values represent the mean ± standard error of the mean. * *p* < 0.05.

A representative image of BALF from each group is shown in Figure 3. In the PBS-challenged mice (Control and IMB groups), the cells in the BALF were almost all macrophages, and there were no changes in the total cell counts and differential cell counts due to ImmuBalance treatment. The total cell count was obviously increased by OVA sensitization and challenge (Figure 4A), mostly due to eosinophils. The number of eosinophils was significantly lower in the mice of the OVA/IMB group compared to those of the OVA group (*p* < 0.05; Figure 4B). The numbers of neutrophils and lymphocytes were also significantly lower in the mice of the OVA/IMB group compared to those of the OVA group (Figure 4C,D). However, the number of macrophages did not change (Figure 4E).

### 3.3. ImmuBalance Regulated the Th1- and Th2-Related Cytokines in BALF

**Figure 5:**
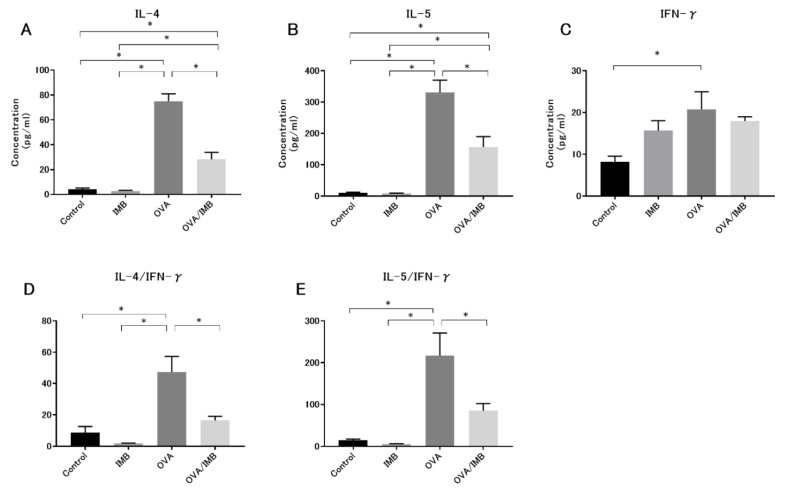
The levels of cytokines in BALF. The levels of IL-4 and IL-5, the ratio of Th2 to Th1 cytokines were significantly attenuated by ImmuBalance treatment (**A**,**B**,**D**,**E**), but no significant change was observed in the levels of IFN-γ (**C**). Values represent the mean ± standard error of the mean. * *p* < 0.05.

The levels of IL-4 and IL-5 in BALF were markedly higher in the OVA and OVA/IMB groups compared to the Control and IMB groups (*p* < 0.05). Comparison of the OVA and OVA/IMB groups showed that ImmuBalance treatment inhibited the levels of these Th2 cytokines (*p* < 0.05; Figure 5A,B). There was no significant difference in the IFN-γ level between the groups with or without ImmuBalance treatment (Figure 5C); however, there was a tendency for the IFN-γ level to increase in the IMB group compared to the Control group, and the ratio of Th2 to Th1 cytokines clearly decreased by ImmuBalance treatment (*p* < 0.05; Figure 5D,E).

### 3.4. ImmuBalance Ameliorated OVA-Induced Histological Changes in Lung

**Figure 6:**
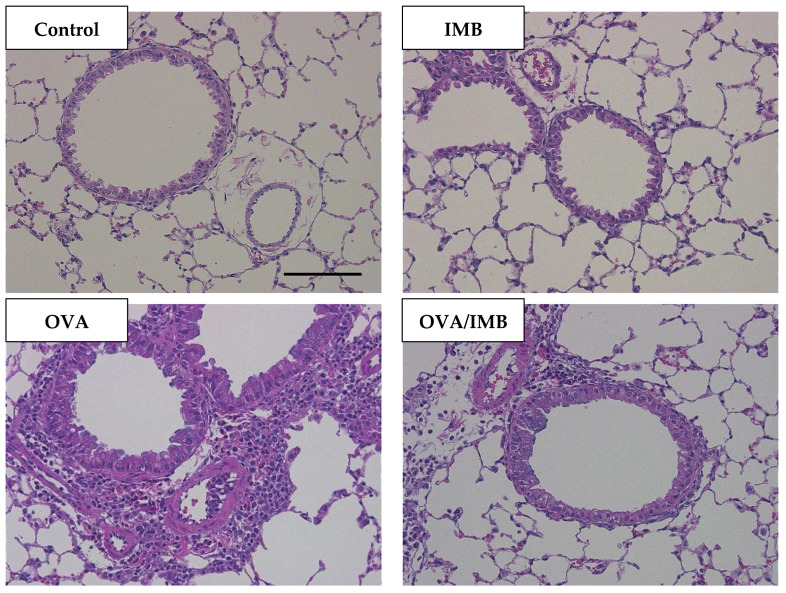
Representative images of lung tissues by HE staining from each group are shown at 200× magnification. Scale bar = 100 µm.

**Figure 7:**
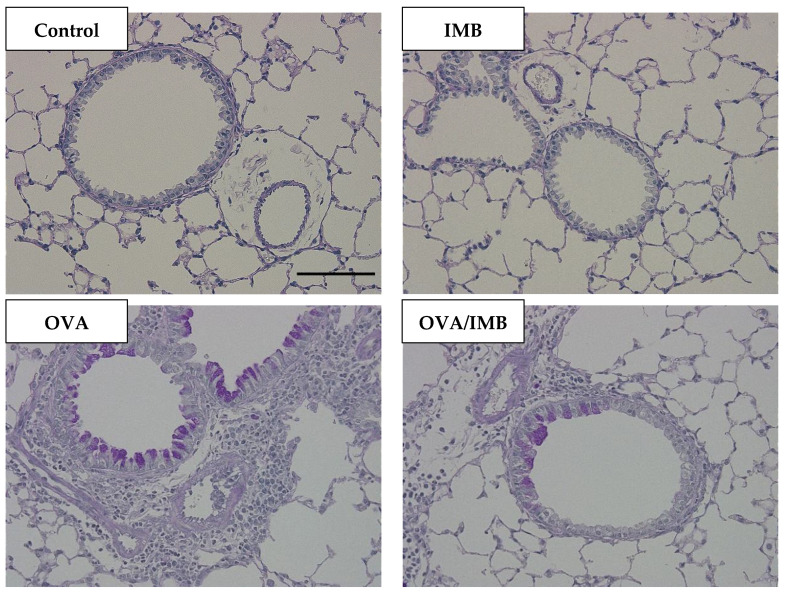
Representative images of lung tissues by PAS staining from each group are shown at 200× magnification. Scale bar = 100 µm.

**Figure 8:**
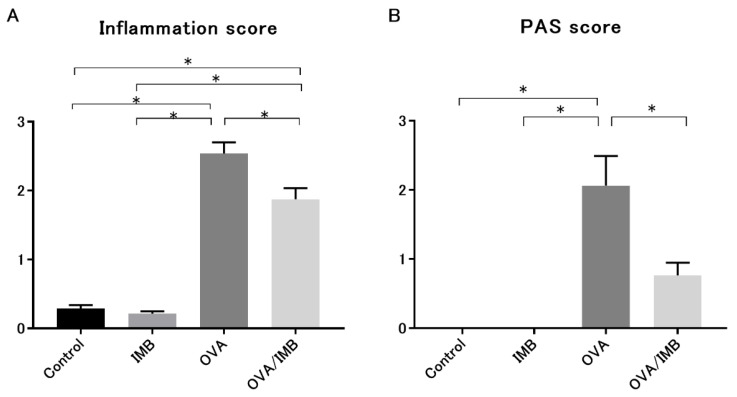
Histological examination of lung tissues. Lung inflammation was evaluated by HE staining, and hyperplasia of goblet cells was assessed by PAS staining. The semi-quantitative scores of inflammatory infiltration and goblet cell hyperplasia were significantly lower in the OVA/IMB group than in the OVA group (**A**,**B**). Values represent the mean ± standard error of the mean. * *p* < 0.05.

A representative image of lung tissue from each group is shown in Figure 6 and Figure 7. OVA sensitization and challenge resulted in peribronchiolar and perivascular inflammation, thickening of the bronchial epithelium, and goblet cell hyperplasia. Peribronchiolar and perivascular inflammation was characterized by the infiltration of eosinophils and lymphocytes. Our semi-quantitative evaluation showed that ImmuBalance treatment significantly reduced inflammatory cell infiltration in the lung (*p* < 0.05; Figure 8A) as well as goblet cell hyperplasia (*p* < 0.05; Figure 8B).

### 3.5. ImmuBalance Reduced the Serum OVA-Specific IgE Levels in OVA-Challenged Mice

**Figure 9:**
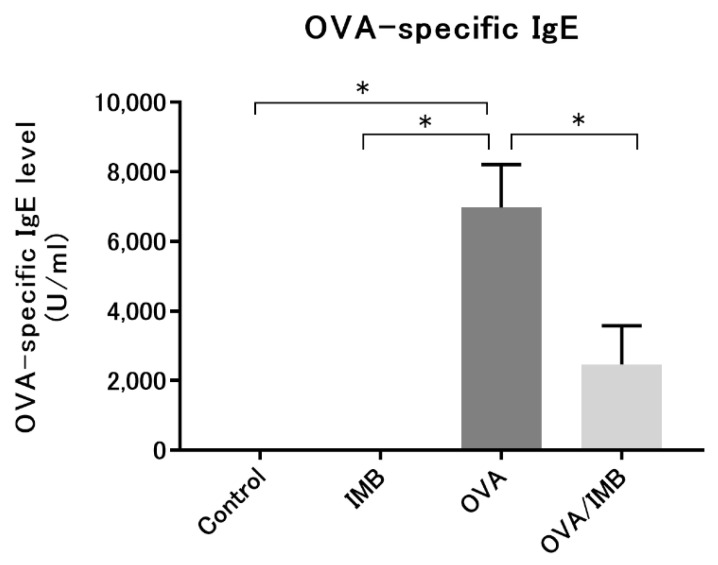
Serum IgE levels were significantly attenuated by ImmuBalance treatment. Values represent the mean ± standard error of the mean. * *p* < 0.05.

Serum OVA-specific IgE was not detected in the Control and IMB groups, while in the OVA and OVA/IMB groups, it was detected at significantly elevated levels, indicating that sensitization by OVA was successful. This elevation in the serum OVA-specific IgE level was abolished by ImmuBalance treatment (*p* < 0.05; Figure 9). However, ImmuBalance treatment did not significantly affect the concentration of SCFAs in the cecum (data not shown).

## 4. Discussion

In this study, we demonstrated that supplementation with the fermented soy product ImmuBalance ameliorated allergic airway inflammation, including the infiltration of inflammatory cells and mucus hypersecretion in a murine model of OVA-induced asthma. In addition, we showed that ImmuBalance suppressed the Th2 cytokine levels in BALF as well as OVA-specific IgE production in serum. The level of IFN-γ, which is a Th1 cytokine, was not elevated by ImmuBalance administration; however, the ratio of Th2 cytokines to IFN-γ clearly decreased. Elevated levels of Th2 cytokines, such as IL-4 and IL-5, play an important role in allergic airway inflammation. IL-4 may lead to eosinophilic inflammation in the lungs and enhance IgE production [26], and IL-5 plays a key role in the activation and survival of eosinophils [27]. Our results suggest that ImmuBalance ameliorates eosinophilic airway inflammation by regulating the immune system, especially by suppressing the secretion of Th2 cytokines.

Soy, the raw material for ImmuBalance, is a basic food ingredient and a common solution for nutritional issues due to its high protein content. It is rich in important nutritional components, such as carbohydrates, vitamins, minerals, saponins, isoflavones, flavonoids, and peptides [28]. In particular, soy isoflavones are estrogenic polyphenols found in soybeans, and they have been reported to have anti-inflammatory effects [29,30,31]. An association between soy consumption and allergic diseases has been suggested [9,10,32]. However, soy is among the most common foods that cause allergies, in addition to other protein-rich foods, such as eggs, wheat, and milk. Sixteen different allergens were identified in soybeans, and the major allergens were Gly m Bd 30K, Gly m Bd 28K, and Gly m Bd 68K [33].

Fermentation techniques have long been used to preserve food. Recently, they have been applied to enhance the nutritional and functional properties of food compared to raw materials and to confer health benefits. Fermentation of soy with a variety of microorganisms increases the amount of isoflavones and improves its nutritional value due to the high concentrations of produced aglycones, minerals, and amino acids [34,35]. In addition, the fermentation of soybean with *A. oryzae*, a microbe used to ferment various traditional legume foods in Japan, resulted in reduced immunoreactivity and eliminated antigenic soybean proteins [36]. Therefore, the fermentation of soy not only enhances its nutritional value and functionality but is also considered to be safe.

The fermented soy product ImmuBalance is already available as a dietary supplement. In a report on the compositional analysis of ImmuBalance in comparison to the raw material (defatted soybeans), the amounts of Gly m Bd 30K and Gly m Bd 28K were clearly reduced and the amounts of aglycones were increased via glycoside degradation [37]. In this study, we hypothesized that the consumption of ImmuBalance would increase the concentration of SCFAs, especially butyrate, in the intestinal tract and result in the suppression of eosinophilic airway inflammation. However, despite the suppression of eosinophilic inflammation, analysis of the levels of SCFAs in the cecum revealed no significant difference between the mice fed the standard diet and those fed the ImmuBalance-containing diet. This may be due in part to the death or inactivation of probiotics, such as lactic acid bacteria, from the radiation sterilization, and that the difference in the dietary fiber content between the standard diet and the ImmuBalance-containing diet was small. The effects of ImmuBalance on some allergic diseases in murine models have been reported [21,22]; however, the mechanisms were not clearly demonstrated. On the other hand, daidzein, a common isoflavone found in soybeans, was reported to have anti-allergic effects [38].

The results of this study and those of previous reports suggest that the airway inflammation-suppressing effect is not solely due to the substances contained in soybeans, and that the components produced by fermenting soybeans, such as isoflavones, may be important. A randomized clinical trial showed that supplementation with soy isoflavones in patients with poorly controlled asthma did not improve lung function or clinical outcomes [39]; therefore, the therapeutic effect of soy components on asthma has not been proven. On the other hand, questionnaire-based studies have reported an association between soy intake and improved lung function in asthma patients [9,32]. However, these studies did not assess actual airway inflammation. In our study, ImmuBalance administration was started at the beginning of the experiment, not after the asthma model was induced. ImmuBalance decreased not only the secretion of Th2 cytokines but also IgE production, indicating that it has a preventive effect rather than a therapeutic effect on asthma. We also histologically assessed airway inflammation; therefore, our results may complement those of previous clinical studies. In addition, the effect of ImmuBalance on peanut allergy was already reported [22], but our results suggest that ImmuBalance may be effective for a wider range of allergic diseases. This is because peanut allergy is completely dependent on IgE and is classified as a type I allergy, whereas asthma involves many other factors in addition to IgE, and is not a type I allergy. Identification of the active components responsible for the anti-inflammatory effects of ImmuBalance will help determine and expand its clinical potential for the prevention or treatment of allergic diseases.

This study has some limitations. Firstly, we used an acute asthma model; therefore, we could not reproduce chronic airway inflammation and remodeling of the airways. Secondly, we could not test for airway hyperresponsiveness. Thus, we could not state definitively that ImmuBalance improved the pathogenesis and clinical outcomes such as lung function or symptoms of asthma. Further research is required to clarify these points. In conclusion, we demonstrated that supplementation with the fermented soy product ImmuBalance ameliorated allergic airway inflammation in a murine model of OVA-induced asthma.

## Data Availability

The data reported in this study are available on reasonable request from the corresponding author.
